# CDX-301: a novel medical countermeasure for hematopoietic acute radiation syndrome in mice

**DOI:** 10.1038/s41598-020-58186-1

**Published:** 2020-02-04

**Authors:** Merriline Satyamitra, Lynnette Cary, Dylan Dunn, Gregory P. Holmes-Hampton, Lawrence J. Thomas, Sanchita P. Ghosh

**Affiliations:** 1 Radiation and Nuclear Countermeasure Program, DAIT, NIAID, 5601 Fishers Lane, Rockville, MD 20892 USA; 2 Armed Forces Radiobiology Research Institute, Uniformed Services University of the Health Sciences Bethesda, Bethesda, MD 20889 USA; 3 Celldex Therapeutics, Inc., Needham, MA 02494 USA

**Keywords:** Biochemistry, Drug discovery, Physiology, Signs and symptoms

## Abstract

Bone marrow failure and hematopoietic damage is one of the major consequences of irradiation-induced lethality. There is an immediate need to develop medical countermeasures (MCMs) to combat irradiation-induced lethality. We tested the efficacy of CDX-301, developed by Celldex Therapeutics Inc., in mice exposed to Co-60 gamma total body irradiation (TBI). The drug demonstrated its efficacy both as a prophylactic countermeasure and a mitigator in CD2F1 mice exposed to TBI. A single dose of CDX-301 administered 24 h prior to 24 h post–exposure conferred significant survival. Accelerated recovery from irradiation-induced peripheral blood cytopenia, bone marrow damage as well as apoptosis in sternum was observed in mice pre-treated with CDX-301. Analysis of splenocytes revealed alterations in T cell profiles that were dependent on the time of drug administration. Prophylactic treatment of CDX-301 resulted in increased splenic CD3+ T cells, specifically CD4+T helper cells, compared to splenocytes from non-irradiated mice. These results indicate that CDX-301 is a promising radiation countermeasure and demonstrate its capability to protect cells within hematopoietic organs. These data support potential use of CDX-301, both pre- and post-radiation, against hematopoietic acute radiation syndrome with a broad window for medical management in a radiological or nuclear event.

## Introduction

In today’s world, there is an unmet need for medical countermeasures against radiation due to increased radiation threats, and in particular, those medical countermeasures which have a broad window of medical management. Following a radiological scenario (accidental or deliberate), one should expect substantial delays in delivering medical care to the affected population. Radiation countermeasures which can be used either before or shortly after exposure, are required to protect the first responders deployed for rescue operations. Although efforts to identify and develop radiation countermeasures for acute radiation syndrome (ARS) were initiated decades ago^1–3^, there are only three FDA approved radiation countermeasures available to treat patients as early as 24 h after exposure: Neupogen, Neulasta, and Leukine^4–6^. A situation may arise that medical countermeasures will be needed for military and first responders 24 h before going for rescue operations, as well as shortly after they reach the contaminated field. This situation has prompted increased research efforts to identify a new generation of medical countermeasures against radiation, which can be administered both before and after exposure.

**Figure 1:**
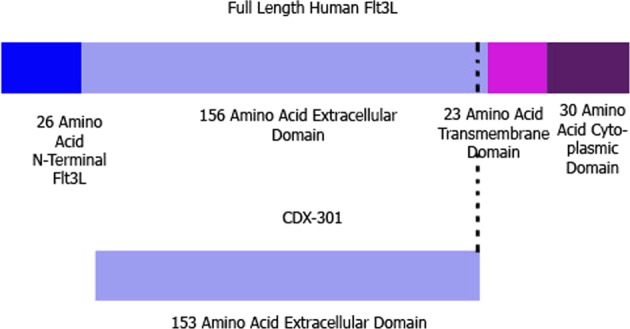
Structure of CDX-301. Representative image of CDX-301 molecule (bottom), a 153 amino acid domain from the human Flt3 ligand (Flt3L) (top) lacking the N-terminal segment and c-terminal region along with the transmembrane domain.

CDX-301 is the soluble recombinant human protein form of the Fms-related tyrosine kinase 3 ligand (Flt3L), a hematopoietic cytokine. CDX-301 is a non-covalent homodimer of a recombinant, soluble glycoprotein which consists of the 153 N-terminal amino acids of the human Flt3L extracellular domain (Fig. 1). CDX-301 enhances immune cell reconstitution in mice^7^, and acts on both myeloid and lymphoid lineages to overcome long-lasting lymphoid cell deficiencies that may occur during the acute radiation syndrome (ARS). Preclinical studies have demonstrated that human CD34+ Flt3+ HSC are capable of reconstituting myelopoiesis and, in particular, lymphopoiesis *in vivo* in NOD/SCID mice^8^, indicating the potential of Flt3L to overcome long lasting lymphoid deficiencies associated with radiation exposure. Toxicology studies show that the drug is well tolerated, and a Phase 1 Clinical Trial was completed with the goal of marketing this product for hematopoietic stem cell transplantation^9^ and cancer immunotherapy^10^, providing evidence of the interest to move this drug quickly from the bench to the clinic as a marketable product.

The expression of the Flt3 receptor (CD135) is restricted to hematopoietic stem and progenitor cells (HSPC), immature thymocytes, and steady state dendritic cells (DC)^11^. Flt3L binding induces receptor dimerization which activates signaling pathways involved in cell survival, proliferation, and differentiation. The mobilization of CD34+ stem and progenitor cells motivated the initial clinical trials of Flt3L in autologous hematopoietic stem cell transplantation (HSCT) in breast cancer patients and in non-Hodgkin lymphoma or ovarian cancer patients. A combination therapy of rhuFlt3L with GM-CSF or G-CSF led to sustained higher levels of CD34+ and colony forming hematopoietic progenitor cells the G-CSF alone^9,12^.

During HSCT the immune system is slow to recover and in fact may not fully do so^13,14^, mobilization of Flt3 receptor expressing HSCs, myeloid and lymphoid pregenitors, DCs and immature thymocytes are critical for successful allogenic HSCT^15,16^. Neutrophils and natural killer (NK) cells recover quickly while donor DC competence is delayed diminishing the capacity for B and T cell immune reconstitution following HSCT which accounts for much of the late morbidity and mortality from increased opportunistic infection and malignant relapse. Flt3L enhanced thymic-dependent and thymic-independent immune reconstitution in mice^7^. Flt3 signaling is involved early on in the generation of lymphoid lineage precursors from multipotent hematopoietic progenitors (MPP) in bone marrow^17^, indicating the potential of Flt3L to overcome long lasting T cell deficiencies. Since ionizing radiation (IR) damages the hematopoietic system, this function of Flt3L may prove extremely beneficial where both myeloid and lymphoid cells in circulation and in tissue are affected, and lymphoid cells, in particular, exhibit slow recovery. The current paper describes the efficacy of the CDX-301 molecule as a prophylactic as well as a mitigator in mice exposed to whole body gamma radiation and provides evidence that the effects of CDX-301 include protection and recovery of both myeloid and lymphoid cells in circulation and within tissue. Interestingly, the recovery of lymphoid cells within tissue from irradiated mice vary depending on the time of drug administration.

## Methods

### Mice

Inbred CD2F1 male mice 8–9 weeks of age were sourced (Envigo Corporation, Indianapolis, IN, USA) and housed in the AFRRI vivarium in conditions described previously^18^. Briefly, animals were examined by veterinary staff and determined to be free of adventitious agents and pathogens, including Pseudomonas aeruginosa, Klebsiella pneumonia and Pasteurella. Mice were free of Sendai, pneumonia virus of mice (PVM), reovirus-3 (Reo 3), mouse adenovirus (MAD-1, MAD-2), mouse cytomegalovirus (MCMV), Ectromelia, K virus, lymphocytic choriomeningitis virus (LCM), epidemic diarrhea of infant mice (EDIM), Hantaan virus, rotavirus, mouse parvovirus (MPV), polyoma virus, mouse minute virus (MMV), mouse thymic virus (MTV), Theiler’s mouse encephalomyelitis virus (TMEV/GDVII), Encephalitozoon cuniculi, CAR bacillus, Mycoplasma pulmonis and Clostridium piliforme. AFRRI’s Veterinary Science Department determined the mice were endoparasite- and ectoparasite-free. Mice were acclimated in the facility for 2 weeks prior to use in experimental study. Mice were housed in polycarbonate boxes with filter covers (Microisolator, Lab Products Inc., Seaford, DE, 4 per box) with autoclaved Sani-Chips (P.J Murphy Forest Products Corp., distributed by Harlan Teklad) used as bedding material in each cage. Mice had *ad libitum* access to Harlan Teklad Rodent diet 8604 (Purina Mills, St. Louis, MO) and acidified water (pH 2.5–3.0). Rooms housing animals were maintained at a temperature of 21 ± 2 °C, a relative humidity of 50 ± 10%, and 10–15 hourly cycles of fresh air. Animals were maintained in a 12:12 h light:dark cycle using an automated system.

### Irradiation

Irradiation was performed as described previously^19,20^. Unanesthetized mice were irradiated bilaterally at AFRRI’s Cobalt-60 γ-irradiation facility. During irradiation, the animals were placed in well-ventilated plexiglass chambers made specifically for mouse irradiation. The mid-line dose to the animals was delivered at a dose rate of ~0.6 Gy/min. An alanine/ESR (electron spin resonance) dosimetry system (American Society for Testing and Material Standard E 1607) was used to measure dose rates (to water) in the cores of acrylic mouse phantoms. Phantoms were 3 inches long and 1 inch in diameter and were located in all empty compartments of the exposure rack. ESR signals were measured with a calibration curve based on standard calibration dosimeters provided by the National Institute of Standard and Technology (NIST, Gaithersburg, MD). The accuracy of the calibration curve was verified by intercomparison with the National Physical Laboratory (NPL) in the United Kingdom. The only corrections applied to the dose rates in phantoms were for decay of the Cobalt-60 source and for a small difference in mass energy-absorption coefficients for water and soft tissue. The radiation field was uniform within ±2%.

### Ethics statement

All animal procedures were reviewed and approved by the AFRRI Institutional Animal Care and Use Committee (IACUC) using the principles outlined in the National Research Council’s *Guide for the Care and Use of Laboratory Animals*^19,21^. After irradiation animals were returned in their cages and monitored three to four times daily (early morning, late morning, late afternoon and evening). Morbid animals were monitored very closely according to their health in accordance with pre-defined criteria described and approved in the IACUC protocol. Pain and distress were monitored using several criteria including unresponsiveness, abnormal posture, unkempt appearance, immobility, and lack of coordination^22,23^. Mice were considered moribund when they showed an inability to remain upright, were cold, unresponsive or displayed decreased or labored respiration. Moribund mice were euthanized according to American Veterinary Medical Association (AVMA) guidelines.

### Generation of CDX-301 molecule

Human Fms-like tyrosine kinase-3 ligand (Flt3L) is a type I transmembrane glycoprotein comprised of four domains. Natural Flt3L is found as both a cell surface and a released soluble form. Flt3L, manufactured under cGMPs by Celldex Therapeutics (designated CDX-301), is soluble, recombinant human Flt3L in which 3 C-terminal residues of the extracellular domain, the transmembrane domain, and the cytoplasmic domains have all been eliminated. CDX-301 is a glycoprotein with 2 potential N-linked and 5 potential O-linked glycosylation sites. CDX-301 is a polypeptide containing 26 amino acids in the N-terminal domain and 153 amino acids of the extracellular domain. CDX-301 was purified as described previously^9^ from rhuFlt3L expressed in a Chinese hamster ovary cell line using anion exchange, hydrophobic interaction, and cation exchange chromatography. CDX-301 identity, purity, stability, and potency were fully characterized using appropriate GMP-compliant assays.

### CDX-301 formulation for administration

CDX-301 was formulated in normal sterile saline (0.9% NaCl) before use. Saline (0.9% NaCl) was purchased from the Uniformed Services University of the Health Sciences (USUHS) pharmacy and used as vehicle control. Either drug or its vehicle was injected subcutaneously (SC) at the nape of the neck, pre- or post-TBI at the time indicated for each study.

### Fourteen-day acute safety study in mice

To assess the safety of CDX-301, two groups of CD2F1 male mice (n = 6 per group) were SC administered either CDX-301 (1600 μg/kg) or its vehicle saline. The animals were monitored for acute (1 to 4 h) signs of toxicity after administration of drug, then daily for 14 days. Clinical signs of acute toxicity included decreased activity, squinting eyes, hunching, labored breathing or mortality. Weights of the animals were recorded at various intervals during the study. All animals were euthanized on day 14 and a gross necropsy was carried out for any abnormal pathology in all major organs. No changes in behavior (activity, grooming, feeding), or body weight were observed.

### Radioprotection studies

#### Selection of optimum dose for drug administration prior to irradiation

The optimum dose of CDX-301 was identified essentially as described previously^19^. Briefly, groups of 24 mice were injected SC with 0.1 ml vehicle (PBS) or CDX-301 at concentrations of either 400, 800, or 1600 μg/kg 24 h prior to total-body irradiation (TBI) with an estimated LD70/30 (radiation dose that results in the mortality of 70% of mice in 30 days) dose of γ-rays. After exposure, mice were returned to their cages with free access to food and water, and monitored (30 days) for weight loss, apparent behavioral deficit, and survival.

#### Selection of optimum time for drug administration

Based on the findings of the dose optimization experiment and essentially as previously described^19^, a dose of 800 μg/kg CDX-301 was selected for optimizing the time of prophylactic drug administration. Groups of 24 mice were injected SC with 800 μg/kg at 12, 24, 48, or 72 h prior to TBI, and 30-day post-irradiation survival was monitored.

#### Dose reduction factor determination (prophylactic)

Similar to methods used in previous studies^19^, vehicle or 800 μg/kg CDX-301 was administered 24 h prior to TBI. Radiation doses for the vehicle group were 8.0, 8.5, 9.0, 9.5, 10.0, and 10.5 Gy; doses for the CDX-301-treated group were 9.0, 9.5, 10.0, 10.5, 11.0, and 11.5 Gy (dose rate 0.6 Gy/min). The LD50/30 doses for vehicle- and CDX-301-treated mice were generated from the probit plot of survival as a function of radiation dose. The DRF was calculated as the ratio of the LD50/30 dose of drug-treated mice to the LD50/30 for vehicle-treated mice.

### Mitigation studies

#### Drug dose and time optimization

Groups of 24 mice each were irradiated with 9.25 Gy (LD70/30 radiation dose) and injected SC with PBS, or 400, 800, 1600, or 2000 μg/kg CDX-301 4 h after TBI, and 30-day survival monitored. Similarly, mice were irradiated with 9.25 Gy and CDX-301 administered SC 24 h after TBI.

#### Dose reduction factor studies determination (mitigation)

Mice were irradiated with 8.0, 8.5, 9.0, 9.5, 10.0, and 10.5 Gy and injected with PBS 4 h after exposure. The drug group were irradiated with 8.5, 9.0, 9.5, 10.0, or 10.5 Gy and injected with 1600 μg/kg CDX-301, SC 4 h following TBI. DRF was calculated as illustrated in the earlier section.

#### Hematology and histopathology

We used a lower radiation dose (7 Gy) in the hematological studies as this dose is known to induce severe myelosuppression without accompanying lethality, allowing for sufficient sampling numbers after radiation exposure^19,24^. CD2F1 male mice exposed to total body irradiation (TBI) with 7 Gy were euthanized on days 4, 7, 10, 14, 21, and 28 post-TBI. Blood from 8 mice per group per time point was analyzed for CBC with differential as reported using the ADVIA 2120 (Siemens Medical Solutions Diagnostics, Dublin, Ireland), and data were generated using MS software, version 5.9^24^.

Similarly, bone marrow from the sternum was the primary tissue used for histopathologic analysis on 4, 7, and 14 days after 7-Gy irradiation as previously described^25,26^. Processed and longitudinal sections of 5 µm sternum were stained with hematoxylin and eosin. The samples were blinded and submitted for evaluation by a board-certified pathologist. Cellularity was scored from 0% to 100% as a function of total number of cells (Grade 0 = 0% to 10%; Grade 1 = 11% to 33%; Grade 2 = 34% to 66%; Grade 3 = 67% to 100%). Megakaryocytes were evaluated by a semiquantitative analysis of three adjacent high-power (400×) microscopic fields. Megakaryocytes were also scored (inadequate = <1/high power field (hpf), marginally adequate, and adequate >10/hpf) in an average hpf by two individuals. Finally, a subjective estimation of M:E ratio (ratio of myeloid cells to erythroid cells) was used to represent population shifts of the two lineages. Images were captured with an Olympus DP70 camera and imported into Adobe Photoshop (version CS4) for analysis.

#### Colony forming unit assay

The bone marrow of both femurs was collected from CD2F1 mice at days 1, 4, 7, and 14 as previously described^26^. Bone marrow cells were suspended in MethoCult methylcellulose medium (Stem Cell Technologies, Vancouver, BC, Canada) at a concentration of 10^5^ cells/ml. The assay was performed in triplicate. A total of 1.1 ml cell-medium suspension was dispensed per 35-mm cell culture dish. Cell colonies were counted after 12 days by two individuals.

#### TUNEL assay

Terminal deoxynucleotidyl transferase dUTP nick end labeling (TUNEL) assay as described previously^27^ was used to detect apoptosis in sternal bone marrow from 6 mice. DNA strand breaks of apoptotic cells were identified by the ApopTag Peroxidase *In Situ* Apoptosis Detection Kit (Millipore, Billerica, MA) used according to the manufacturer’s instructions. Briefly, the paraffin-embedded sternum sections were prepared as before, unstained sections were deparaffinized in xylene, rehydrated, washed in PBS, incubated with hydrogen peroxide (3.0%) in PBS for 5 min at room temperature to quench endogenous peroxidase, treated with citrate buffer for antigen retrieval for 20 min. DNA fragments present in apoptotic cell are labeled with digoxigenin-nucleotides (terminal deoxynucleotidyl transferase (TdT) enzyme) and then allowed to bind a peroxidase-conjugated anti-digoxigenin antibody followed by with 4′,6-diamidino-2-phenylindole (DAB) as the chromogen. For negative control experiments, the TdT enzyme was not added in the reaction. The positive control contained in the kit (mammary tissue) was used. Slides were counterstained with methyl green (0.5%, IHC, Woodstock, MD).

To confirm the staining results, all tests were carried out in triplicate. Images were captured with an Olympus DP70 camera and imported into Adobe Photoshop (version CS4) for analysis.

The sections were examined under a light microscope at different magnifications. Representative images were captured with a digital camera.

### Analysis of T cells isolated from lymphocytes by flow cytometry

#### Splenocyte isolation

Spleens (6/group) were removed and dissociated similar to previous methods^28^ however a mechanical stomacher (Seward Laboratory Systems Inc. Bohemia, NY) was used to make cell suspensions. Red blood cells were lysed with ACK lysing buffer (Lonza, Inc. Walkersville, MD) according to manufacturer’s protocol, and splenocytes were washed twice with PBS. Viable cells were counted using a hemocytometer prior to staining for flow cytometry.

#### Flow cytometry

Cell number and viability for all groups were determined by trypan blue exclusion. 1–1.5 × 10^6^ cells/sample were used for staining. Fluorescently labeled antibodies to cell surface markers (cluster of differentiation (CD)) were used to identify specific cell populations. Antibodies used for T cell identification were CD4-PE, CD3-APC, and CD8-FITC. CD11b-APC was used to identify granulocytes/monocytes/macrophages. CD49-PE was used to identify natural killer (NK) cells, and CD69-FITC was used to determine the activation status of cell types. Intracellular IL-2 and IFN-γ was detected by intracellular staining of spleen cells with anti-IL-2 or anti-IFN-γ, following the manufacturer’s instructions (BD Biosciences). All antibodies were purchased from BD Biosciences (San Jose, CA, USA) and acquisition was performed using a BD FACS Calibur. Analysis was performed using FlowJo software (Tree Star, Inc. Ashland, OR). Gates and quadrant markers were held constant between different groups and time points.

#### Western blot

Proteins were extracted from cell lysates using RIPA isolation buffer (Thermo Fisher Scientific, Inc). 30 µg of protein was loaded on a 4–15% gradient acrylamide gel, and gel electrophoresis and Western blots were performed using standard techniques^26^. Blots were probed with CD3, CD11b, and CD11c antibodies (Cell Signaling Technologies, Inc. Danvers, MA).

#### Statistical analysis

All survival data were compared using Fisher’s exact test (one-tailed)^29^ and the generalized Savage (Mantel-Cox) procedure^30^ (BMDP Statistical Software Inc.). Probit analyses were done using PASW statistics 18 regression analyses as described^18^. Hematological parameters were graphed as means ± SEM. These data were analyzed by ANOVA. A p value of <0.05 was considered significant.

## Results

### Production of CDX-301 (Flt3 Ligand)

CDX-301 is soluble, recombinant human Ftl3L in which three C-terminal residues of the extracellular domain, the transmembrane domain and the cytoplasmic domains have all been eliminated (Fig. 1). The mature, secreted protein is thus 153 amino acids with a predicted polypeptide molecular weight of 17,445 Daltons, and an apparent molecular weight of 23,000 ± 5000 Daltons, by SDS-PAGE. CDX-301 is a glycoprotein with 2 potential N-linked (N100, N123) and 5 potential O-linked (S136, S137, T138, S144, T151) glycosylation sites. There are 3 intramolecular disulfide bonds and the predominant soluble form is a non-covalent homodimer with an apparent molecular weight of approximately 44,000 Daltons by size exclusion chromatography. The amino acid sequence of the molecule is TQDCSFQHSPISSDFAVKIRELSDYLLQDYPVTVASNLQDEELCGGLWRLVLAQRWMERLKTVAGSKMQGLLERVNTEIHFVTKCAFQPPPSCLRFVQTNISRLLQETSEQLVALKPWITRQNFSRCLELQCQPDSSTLPPPWSPRPLEATAP.

### Non-clinical toxicology

Two GLP-compliant toxicology studies have been conducted using CDX-301, one in Cynomolgus monkeys and a second in rats^31^. These studies included 28 daily SC injections in doses ranging from 5–1600 µg/kg/day. Animals were monitored for a two week period following the last dose at which time tissues were collected for histopathological evaluation. These studies indicated CDX-301 was well tolerated up to a dose of 25 µg/kg/day (determined to be the no observed adverse effect level, NOAEL). Adverse effects amongst the two species were only observed in the highest doses (200 and 1600 µg/kg/day) and included bone marrow hyperplasia, increased injection site inflammation, thymic atrophy, and increased infiltration of mononuclear cells to the spleen, liver, lymphnodes, gastrointestinal tract, gall bladder, and urinary bladder.

A 14 day acute safety study in CD2F1 mice at AFRRI showed no sign of toxicity of CDX-301 administered at a single dose of 1600 µg/kg. Based on these observations, a single dose of 400 to 1600 µg/kg CDX-301 was used in the pre-TBI studies. A higher dose of 2000 µg/kg was used in the post-TBI studies.

### CDX-301 protects against radiation lethality

**Figure 2:**
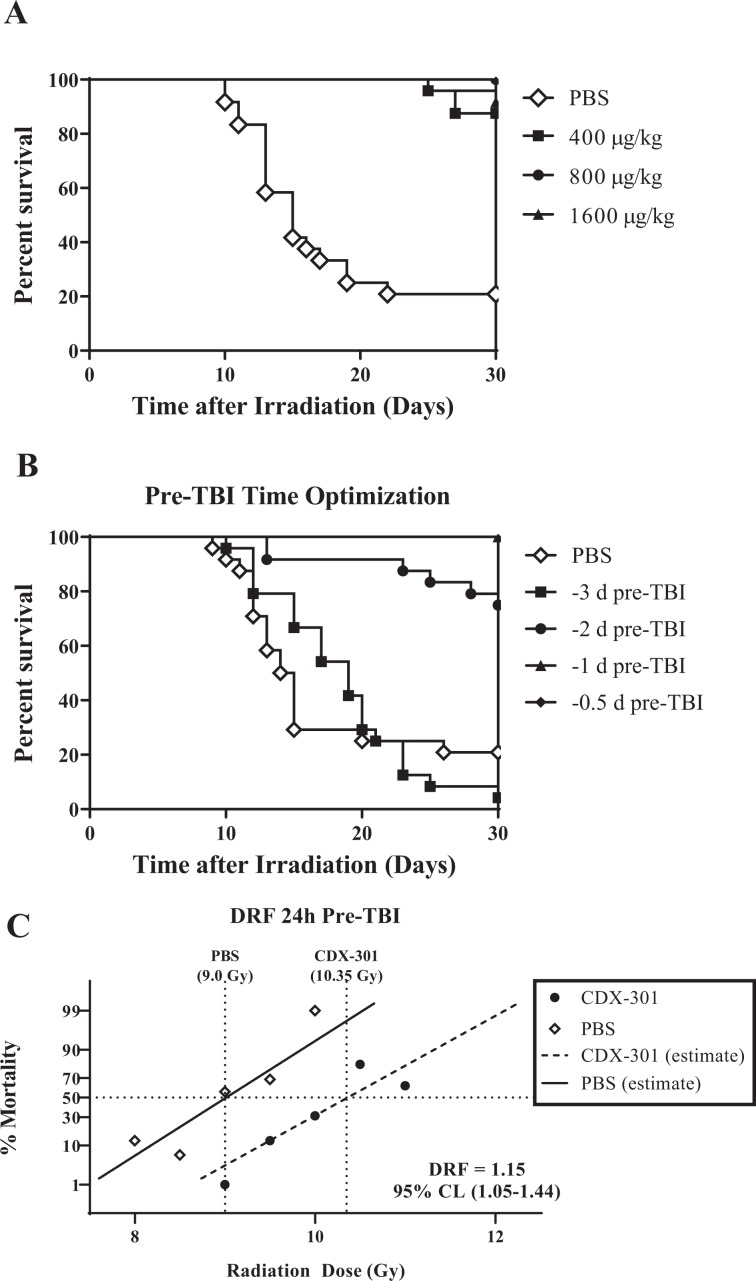
Radiation prophylaxis by CDX-301. Panel A. Survival of CD2F1 mice (n = 24) injected with PBS (◊), 400 (■), 800 (●), or 1600 (▲) µg/kg CDX-301 24 h prior to irradiation with 9.25 Gy γ-rays. Panel B. Time optimization for prophylactic administration of CDX-301. CD2F1 mice (n = 24) were injected with PBS (◊) or 800 µg/kg CDX-301 at 0.5 d (♦), 1 d (▲), 2 d (●), or 3 d (■) before exposure to 9.25 Gy. Panel C. Probit curves for CD2F1 mice (n = 16) injected SC with PBS (◊) or 800 µg/kg CDX-301 (●) and irradiated 24 h later with ^60^Co γ-rays ranging from LD-5/30 to LD-95/30 total body radiation. Dose reduction factor (DRF) was calculated as described under M&M.

In preliminary studies, we evaluated the efficacy of a single dose of CDX-301 administered 24 h prior to total body irradiation (TBI) on 30-d survival. CD2F1 mice (n = 24) injected SC with a single dose of PBS, 400, 800 or 1600 µg/kg CDX-301 were irradiated 24 h later with 9.25 Gy. PBS-treated mice showed inappetance, rough coats, decreased body weight, unsteady gait and hunched posture within 7–9 d post-exposure. Mortality peaked between 10–20 d post-TBI and at the end of 30 days, 22% of the mice survived. Mice pre-treated with CDX-301 looked healthy and did not demonstrate symptoms of radiation sickness. All three doses of CDX-301 improved survival significantly (*p* < 0.001). However, the effect was non dose-dependent; 400 µg/Kg CDX-301 increased 30-d survival by 88%, maximal protection was observed with 800 µg/Kg CDX-301 (100%), while 92% survival was noted for the 1600 µg/Kg CDX-301 treated group (Fig. 2A).

Because first responders may not have access to the countermeasure precisely 24 h before entering a radioactive zone, we determined how early CDX-301 could be administered for efficacy. Mice were injected with a single dose of 800 µg/kg CDX-301 on 3, 2, 1, or 0.5 d prior to lethal irradiation. Drug administered 3 days prior to irradiation delayed mortality, but did not improve 30 d survival (4% survival in drug-treated group compared to 22% survival in vehicle group; Fig. 2B). CDX-301 administered 2 d before TBI increased survival to 75%, while 24 h and 12 h pre-TBI resulted in 100% survival of the irradiated mice (*p* < 0.001) (Fig. 2B).

Dose reduction factor (prophylaxis): Fig. 2C summarizes the probit analyses of groups of mice (n = 16) vehicle or CDX-301-treated (800 µg/kg) 24 h prior to irradiation with a range of radiation doses resulting in 5–95% mortality over a 30 d period. CDX-301 pre-treatment shifted the curve to the right, increasing LD50 to 10.35 Gy compared to LD50 of 9 Gy for the PBS-treated group. DRF was estimated to be 1.15 (CI = 1.05–1.44) (*p* < 0.001).

### CDX-301 mitigates radiation lethality

**Figure 3:**
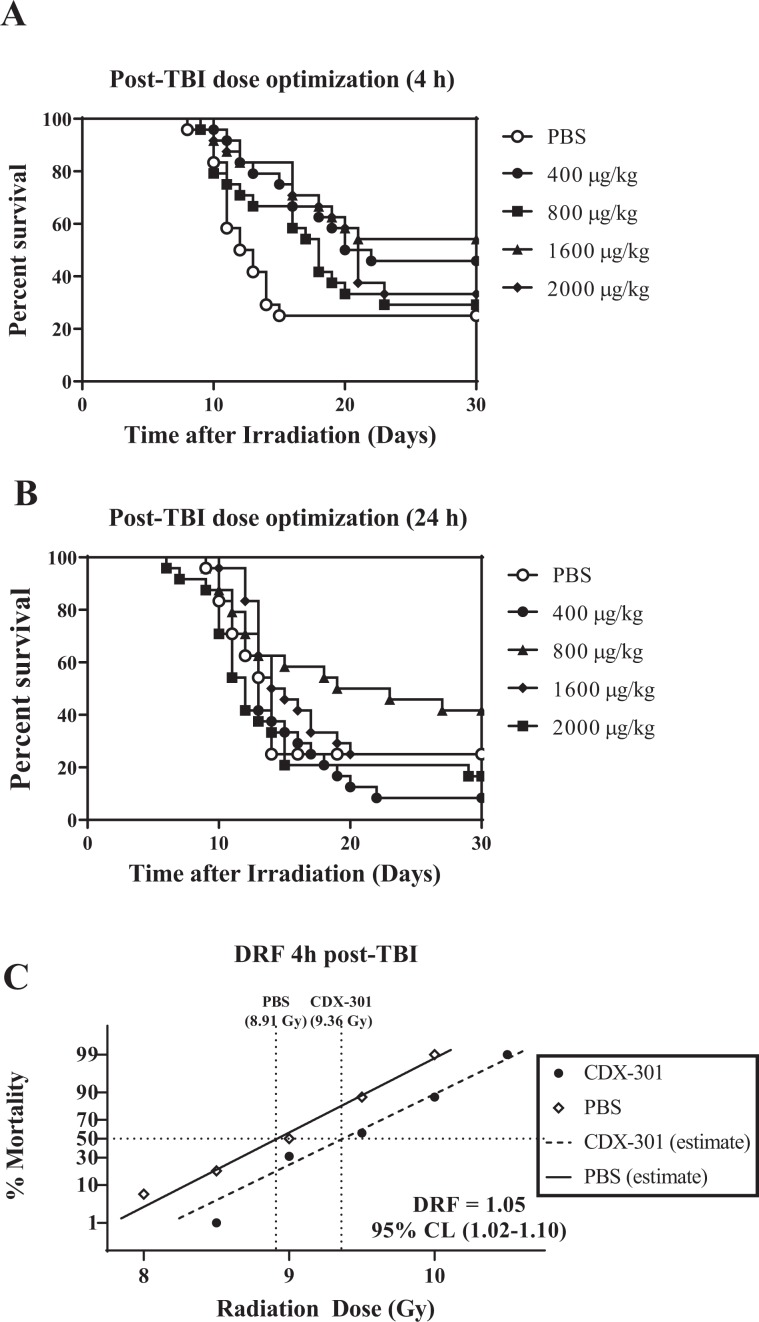
Mitigation of radiation lethality by post-exposure treatment with CDX-301. Panel A. Improvement in 30-day survival of CD2F1 mice (n = 24) irradiated with 9.25 Gy and injected SC 4 h later with PBS (○), 400 (●), 800 (■), 1600 (▲), or 2000 (♦) µg/kg CDX-301. Panel B. Survival of CD2F1 mice (n = 24) irradiated with 9.25 Gy and injected 24 h later with PBS (○), 400 (●), 800 (▲), 1600 (♦), or 2000 (■) µg/kg CDX-301. Panel C. Dose Reduction Factor for CDX-301 administered 4 h post-TBI. Probit curves were generated for PBS (◊) or 1600 µg/kg CDX-301-treated (●) mice.

In the event of unanticipated exposure, it is vital that a countermeasure be effective when given after irradiation. To this end, we evaluated the efficacy of CDX-301 in mitigating radiation lethality using a single dose of the drug administered either 4 h or 24 h after TBI with 9.25 Gy. CD2F1 mice (n = 24) were irradiated and injected with 400, 800, 1600, or 2000 µg/kg CDX-301 4 h after exposure (Fig. 3A). Survival was not drug dose-dependent; 1600 µg/kg CDX-301 improved 30-d survival to 54% compared to 22% survival in the PBS group, with 400 µg/kg CDX-301 affording a 24% improvement in survival (46%). This effect was significantly higher than vehicle control (*p* < 0.05). Both 800 and 2000 µg/kg CDX-301 delayed mortality, but did not significantly increase 30 d survival (Fig. 3A).

We further tested the efficacy of CDX-301 when administered as a single dose 24 h after TBI. Again, survival was not drug dose-dependent; 800 µg/kg improved survival to 42% compared to 25% survival in the PBS-treated group; there was significant delay in mortality in the CDX-301 treated group compared to vehicle. The other drug doses did not enhance survival (Fig. 3B).

Dose Reduction Factor (DRF; mitigation): Post-irradiation treated with 1600 µg/kg CDX-301 4 h after TBI resulted in a DRF of 1.05 (95% CI = 1.02–1.10); the LD50 for PBS group was 8.9 Gy which increased to 9.36 Gy with CDX-301 treatment Fig. 3C).

### Hematological response of irradiated mice to CDX-301 intervention

A sub-lethal radiation dose of 7 Gy was used for hematological experiments as this dose has been shown to induce bone marrow myelosuppression without the associated mortalitly of an LD70/30 dose (9.25 Gy)^24^. Using this lower radiation dose led to increased survival at the later post-irradiation times (days 14–28), which allowed a more robust statistical evaluation.

**Figure 4:**
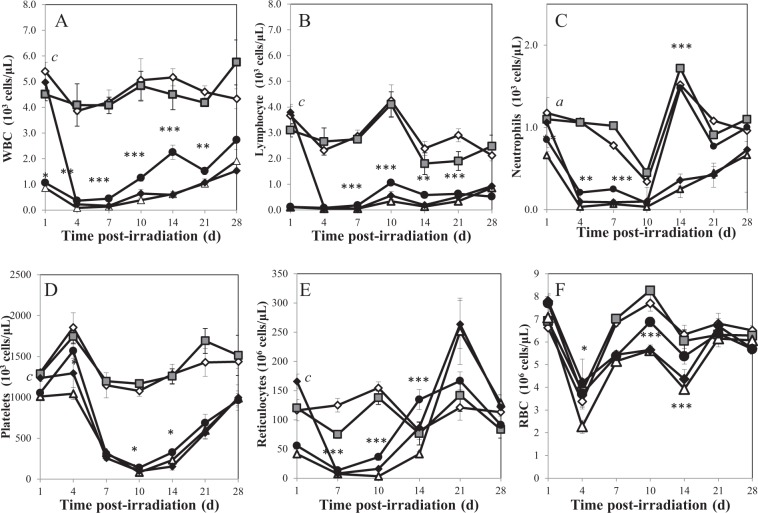
Effect of CDX-301 treatment on peripheral blood counts in 7 Gy-irradiated CD2F1 mice. Mice were treated as follows: 0 Gy (◊,open diamond), 0 Gy CDX-301 (1600 µg/kg, +4 h, ■closed square), 7 Gy TBI (Δ, open triangle), pre-treated with 800 µg/kg CDX-301 (−24 h, ●, closed circle), or post-TBI (+4 h) treated with 1600 µg/kg CDX-301 (♦closed diamond). Panel A: Circulating White Blood Cells (WBC). Panel B: Lymphocytes. Panel C. Neutrophils. Panel D. Platelets. Panel E. Reticulocytes. Panel F. Red Blood Cells (RBC). Data presented are means ± Standard Error of Mean (SEM) from 6 mice. Statistical significance was determined by comparing the irradiated control and CDX-301-treated groups as described. *P < 0.05; **P < 0.01; ***P < 0.001 pre-TBI CDX-301 treated group compared to irradiated control. ^a^*p* < 0.05, ^b^*p* < 0.01, ^c^*p* < 0.001 CDX-301 post-TBI treated group compared to irradiated control.

Irradiation with 7 Gy severely depleted peripheral blood cells (Fig. 4). WBC (Fig. 4A), lymphocytes (Fig. 4B), and neutrophils (Fig. 4C) decreased rapidly from day 1 post-TBI, resulting in a nadir on day 4 post-TBI. These parameters improved gradually on days 14, 21, and 28 post-TBI, but remained significantly lower than sham-treated controls at all times (*p* < 0.001). Furthermore, irradiation with 7 Gy also affected the megakaryocytic and erythropoietic lineages. Platelet counts in the 7 Gy-irradiated group fell to a nadir on day 10 post-TBI and the values were ~7% of the normal controls (82.1 × 10^3^ platelets/µL compared to 1079 × 10^3^ platelets/µL in sham-control). A small increase in platelets was observed on days 14–28 post-TBI in the 7 Gy irradiated group, but these levels remained significantly lower than the naïve control (Fig. 4D). Depletion in reticulocyte levels was also observed on day 10 post-TBI, while RBCs decreased on day 4 and reached a second nadir on day 14 post-TBI. Reticulocytes in the irradiated control preceded RBC recovery with increased reticulocytes counts on day 14 post-TBI to near normal levels and increased profoundly higher than baseline values on day 21 (*p* < 0.001; Fig. 4E). RBC recovery followed a biphasic trend, with a small elevation on day 7 followed by a second nadir on day 14 and complete recovery on day 21 post-TBI (Fig. 4F).

Pre-treatment with CDX-301 abrogated radiation-induced pancytopenia in mouse peripheral blood. Although WBC, lymphocyte, and neutrophil counts were significantly reduced on day 4 post-TBI in the CDX-301 pre-treated group, the counts were significantly higher than the irradiated control (*p* < 0.05–0.01). CDX-301 pre-treatment prevented nadirs for WBC and neutrophils on day 4. This group also demonstrated accelerated recovery of WBC, lymphocytes, and neutrophils on days 7 and 10 post-TBI. Although WBC and lymphocytes were lower than baseline values on day 28, neutrophils were elevated to normal levels by day 14 post-TBI in the CDX-301 pre-treated group. Similarly, CDX-301 pre-treatment prevented nadirs for platelets, reticulocytes and RBC compared to the 7 Gy irradiated group. Further, CDX-301 pre-treatment accelerated recovery of these indices; platelet counts increased significantly on days 10 and 14, as did reticulocyte counts and RBC (*p* < 0.05–0.001). Platelets were not restored to normal values even at the end of the study; however, both reticulocytes and RBCs presented normal profiles day 14 post-TBI (Fig. 4).

Post-irradiation treatment with CDX-301 (4 h after exposure) presented a strikingly different trend. On day 1 post-TBI, all indices in the CDX-301 post-treated group looked similar to normal, sham-treated control, with baseline levels of circulating WBC, lymphocytes, neutrophil, platelets, reticulocytes, and RBC, which were very significantly higher than radiation-alone scores (*p* < 0.001). However, on day 4 post-TBI, these indices plummeted to the levels of the irradiated control, with similar nadirs and counts. Further, recovery of these parameters also mirrored irradiated control for sampling time 4–28 post-TBI, showing no accelerated hematopoietic recovery (data not shown).

### CDX-301 alters morphology of irradiated bone marrow

**Figure 5:**
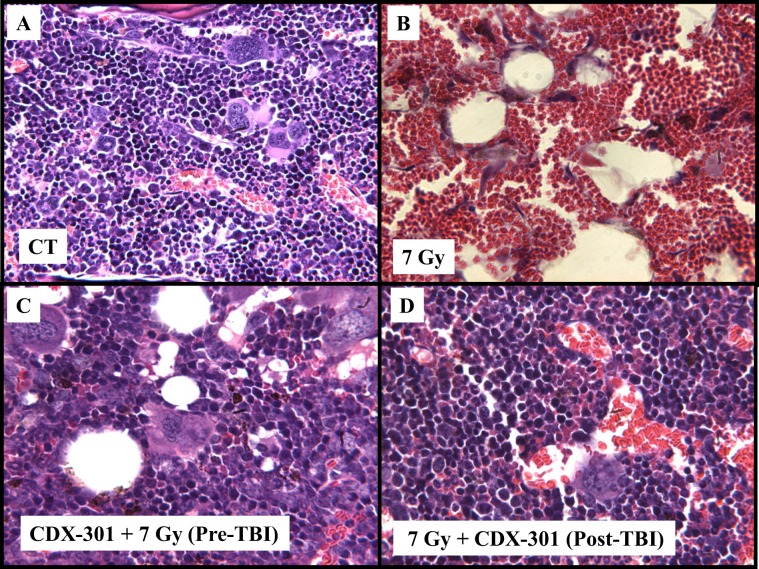
Histopathology. Histological sections of CD2F1 mouse sternum marrow collected from mice 14 days after sham irradiation or 7 Gy irradiation and PBS/CDX-301 treatment. Panel A: Normal sternum marrow histology. Panel B: Mice irradiated with 7 Gy and treated with PBS showing bone marrow atrophy, massive infiltration by adipocytes, and the presence of reactive osteoblasts. Panel C: Irradiated mice pre-treated with CDX-301 showing normal morphology, cellularity, and regenerative megakaryocytes. Panel D: Mice irradiated with 7 Gy and treated with CDX-301 4 h after TBI showing increased megakaryocytes, cellularity, and regenerative morphology. All samples were stained with hematoxylin and eosin (original magnification 200×).

A blinded histological analysis revealed normal sternal bone marrow pathology in the control group (Fig. 5A). However, irradiation at 7 Gy led to severely ablated hematopoietic tissue and hypocellularity (5% in 7 Gy irradiated mice vs. 100% in controls), a shift in the M:E ratio of 10:1 was observed, a reduction in the number of megakaryocytes to 1/high-power field, as well as a striking increase in the number of adipocytes, amount of cell debris, and number reactive osteoblasts in the sternal stroma, also increased sinusoidal blood congestion was noted (day 14 post-TBI in representative image, Fig. 5B). Pre-treatment with CDX-301 24 h prior to irradiation ameliorated the decrease in cellularity (85% in CDX-301 treated vs. 5% in vehicle), increased the number of megakaryocytes to 20/HPF (from 1/HPF) and restored the stromal appearance day 14 post-TBI (Fig. 5C). Day 14 post-TBI analysis of animals treated with CDX-301 post-irradiation also demonstrated improved bone marrow cellularity (45% of sham-irradiated control) and increased megakaryocyte numbers ~8/HPF (Fig. 5D).

### Response of irradiated hematopoietic progenitor cells to CDX-301

**Figure 6:**
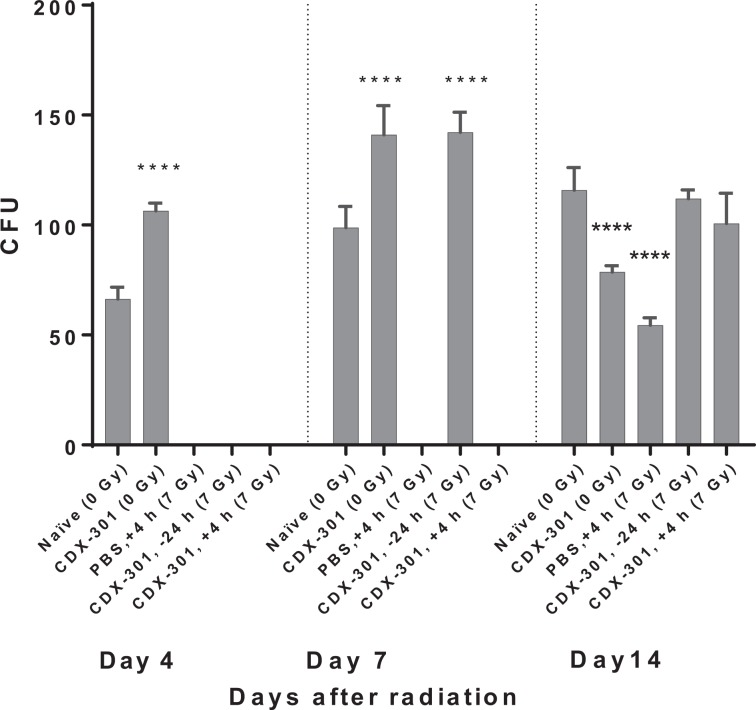
CDX-301 accelerates recovery of hematopoietic progenitor after 7 Gy irradiation. Mice (n = 6) were treated as follows: Naïve (0 Gy), CDX-301(1600 µg/kg, 0 Gy), 7 Gy TBI (PBS, +4 h), pre-treated with 800 µg/kg CDX-301 (−24 h), or post-TBI (+4 h) treated with 1600 µg/kg CDX-301. Colony forming units (CFU) were assayed on days 1, 4, 7, and 14 after exposure from different groups. Day 1 data is not shown since there were no colonies in the irradiated groups. Cells from two femurs were pooled, counted, and each sample plated in triplicate to be scored 12 days after plating. Data are expressed as means ± SEM. Statistical significance was determined between irradiated control and CDX-301-treated group as described. ****P < 0.0001 pre-TBI and post-TBI CDX-301 treated group compared to irradiated control.

To evaluate the number of functional hematopoietic progenitor cells (HPCs) in bone marrow of irradiated mice treated with CDX-301, we next performed the colony forming unit (CFU) assay. Irradiation with 7 Gy reduced proliferation of progenitor cells on days 1, 4, 7, and 14 post-TBI; in fact, no colonies were evident from bone marrow cells plated on days 4 and 7 post-TBI. Pre-treatment with CDX-301 24 h before exposure resulted in a rapid recovery of proliferating progenitor cells seen on day 7 post-TBI. Post-treatment with CDX-301 4 h after exposure to 7 Gy resulted in a dramatic increase in the number of CFU on day 14 post-TBI (Fig. 6). However, there were virtually no colonies remaining on day 4 and 7 post-TBI.

### CDX-301 abrogates irradiation-induced TUNEL positive cells in bone marrow sternum

**Figure 7:**
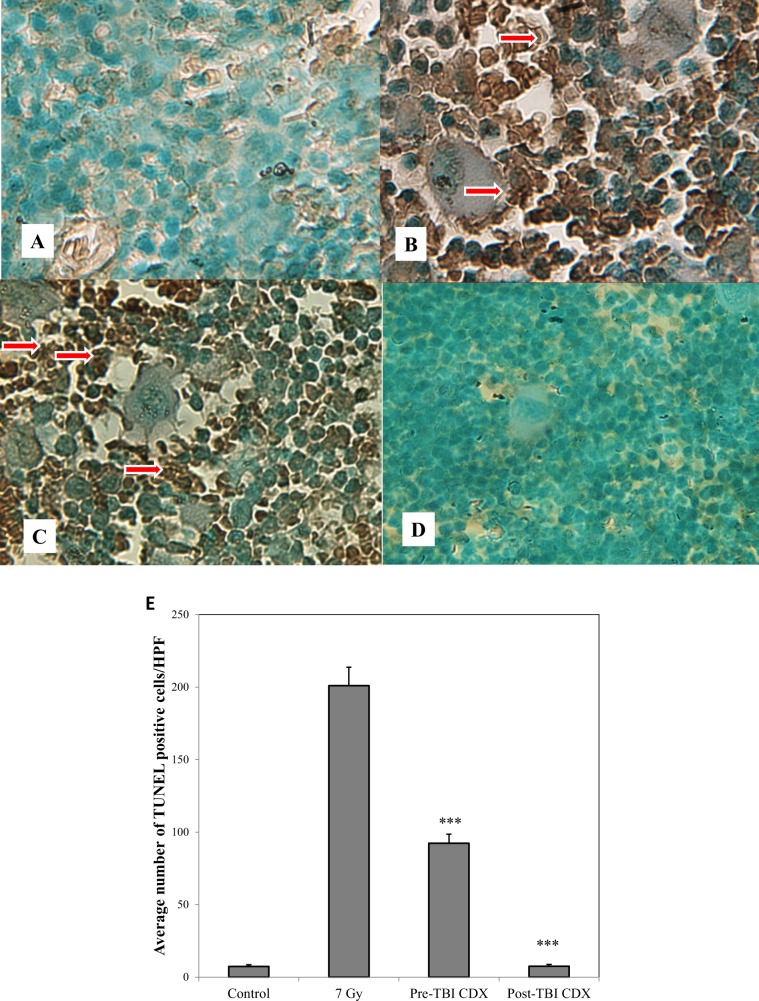
Modulation of apoptosis in bone marrow sternum of 7 Gy-irradiated mice by CDX-301 24 h after exposure. Representative images at 400x magnification are from CD2F1 mice (n = 6) were treated as follows: Panel A. Sham-irradiated. Panel B. 7 Gy irradiated. Panel C. Pre-treated with CDX-301 and irradiated with 7 Gy 24 h after drug administration. Panel D. Irradiated with 7 Gy and injected with CDX-301 4 h after exposure. Panel E. Quantification of TUNEL positive cells under different treatment conditions. TUNEL positive cells are stained deep brown, with accompanying nuclear fragmentation (white and red arrows); all sections are counterstained with methyl green. Data plotted are means ± SEM. Statistical significance was determined between irradiated control and CDX-301-treated group as described. ***P < 0.001 pre-TBI and post-TBI CDX-301 treated groups compared to irradiated control.

Because apoptosis is one of the main routes of radiation-induced clastogenicity, we investigated the efficacy of CDX-301 on modulating this parameter. As expected, irradiation with 7 Gy resulted in severe apoptosis of the highly proliferative bone marrow compared to control (Fig. 7A). The TUNEL positive cells averaged to the excess of 175 cells/HPF on day 1 post-TBI (Fig. 7B,E). We were unable to score the later days post-TBI due to destruction of the bone marrow and unavailability of scorable myeloid or erythroid progenitors. Pre-treatment with CDX-301 did not completely prevent apoptosis, however the number of apoptotic cells was significantly lower than the irradiated control (Fig. 7C,E). In contrast, post-irradiation treatment with CDX-301 was virtually identical to non-irradiated sham-control with ~8 TUNEL positive cells, significantly lower than irradiated control (Fig. 7D,E).

### CDX-301 administration after IR preserved splenic T cells and reduced radiation-induced intracellular cytokine expression

**Figure 8:**
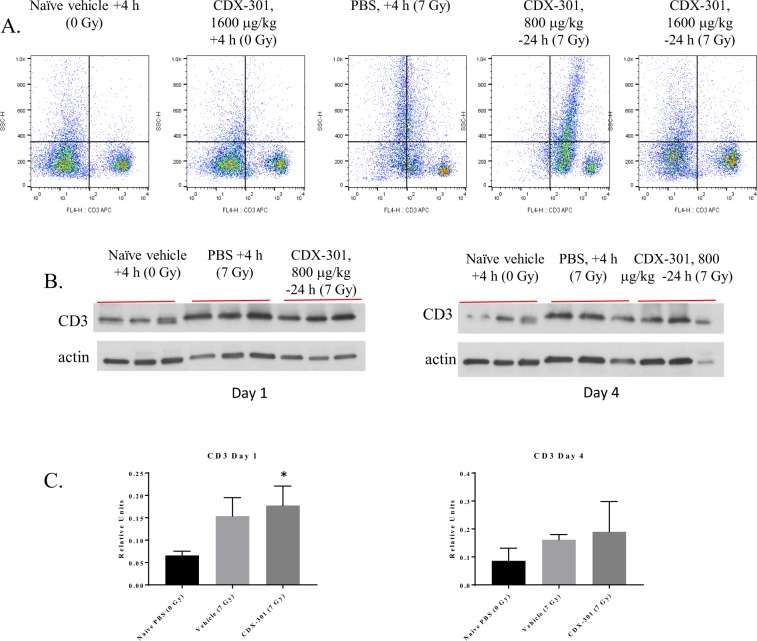
Protection of splenic T-cells by CDX-301. CDX-301 was administered 24 h prior to or 4 h after 7 Gy γ-radiation. The percentage of CD3+ T cells 24 h (day 1) after radiation were detected by flow cytometry (**A**). CD3+ T cell presence in splenocytes treated 24 h prior to radiation was confirmed by Western blot on days 1 and 4 after radiation (**B**) with accompanying densitometry (**C**) *p < 0.02.

**Figure 9:**
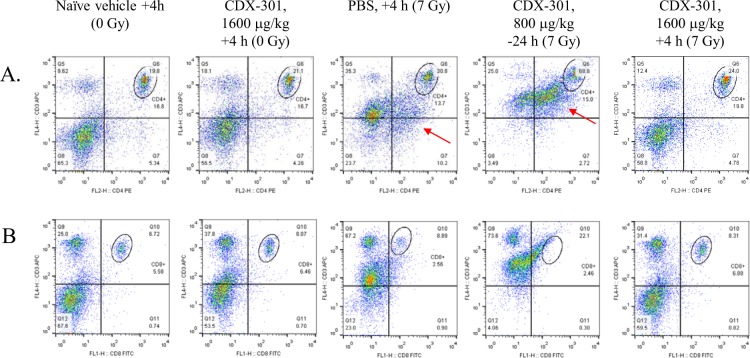
Changes in splenic T cell expression vary with CDX-301 time of administration. CDX-301 was administered 24 h prior to or 4 h after 7 Gy γ-radiation. The percentage of CD4+ CD3+ Th (**A**) and CD8+ CD3+ Tc (**B**) were determined by flow cytometry (circled). A subset of cells with lower CD4+ CD3+expression was identified (red arrow).

**Figure 10:**
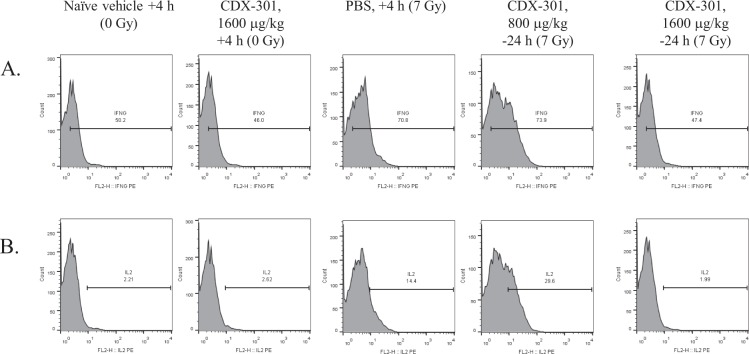
Intracellular IFN-γ (**A**) and IL-2 (**B**) induction in splenocytes was determined by flow cytometry. CDX-301 was administered 24 h prior to or 4 h after 7 Gy γ-radiation. A right peak shift compared to the left-most panel (Naïve vehicle +4 h) indicated increased cytokine expression.

Flow cytometric analysis of spleen cells at day 1 after TBI demonstrated an alteration in overall T cell profiles (Fig. 8A). Similar percentages of T helper (Th) cells, identified as CD4 + CD3+ cells remained in all groups (Fig. 9A, Table 1). Spleens from IR + Vehicle and IR + CDX-301 administered 24 h prior to radiation also contained a population of cells that stained slightly dimmer for both CD4 and CD3 (Fig. 9A, red arrows), noted here as CD4^lo^ CD3^lo^. This population was not seen in splenocytes from irradiated mice administered CDX-301 at T = +4 hr (Fig. 9A). The percentage of cytotoxic T cells (Tc) was analyzed using CD8 and CD3 antibodies (Fig. 9B, Table 1). Irradiation dramatically reduced the percentage of CD8 + CD3+ Tc, and CDX-301 offered no protection as a prophylactic (−24 h). However, splenocytes from irradiated mice given CDX-301 as a mitigator (+4 h) contained a Tc profile similar to non-irradiated mice. Taken together, these data indicate that CDX-301 given after IR protects T cells from initial radiation damage (within the first 24 h). In our experiments IR also induced intracellular IL-2 and IFN-γ expression, and this was also seen when CDX-301 was administered 24 hr prior to IR (Fig. 10A,B). However, treatment with CDX-301 4 h after IR maintained cytokine expression levels similar to that of non-irradiated counterparts. The radiation-induced increase in CD3+ splenocytes was confirmed by Western blot in animals that were treated 24 h prior to radiation (Fig. 8B,C). The difference in CD3 expression in CDX-301 treated animals was significant compared to non-irradiated controls on Day 1.

**Table 1:** Percentages of immune cells in spleens is altered by CDX-301 administration.

Condition (Day 1)	% CD4+ CD3+ T cells	% CD8+ CD3+ T cells	% CD11b + macrophages	% CD11b + CD69 + activated macrophages	% CD49 + NK cells
Naïve vehicle + 4 h (0 Gy)	19.8	5.58	7.8	0.97	4.87
CDX-301 + 4 h (0 Gy)	21.1	6.45	8.92	1.47	6.15
PBS + 4 h (7 Gy)	30.8	2.56	47.9	26.9	13.9
CDX-301-24 h (7 Gy)	68.8	1.00	53.5	32.9	15.2
CDX-301 + 4 hr (7 Gy)	24.0	6.68	11.0	1.34	6.22

Table 1 for CDX-301: a novel medical countermeasure for hematopoietic acute radiation syndrome in mice.

CDX-301 was administered 24 h prior to or 4 h after 7 Gy γ-radiation. The percentages of lymphoid (Tc, Th, CD49 NK), and myeloid (CD11b+ macrophages,) cells, as well as CD69 activation was determined by flow cytometry.

### CDX-301 administration after IR preserved splenic macrophage and NK cells

**Figure 11:**
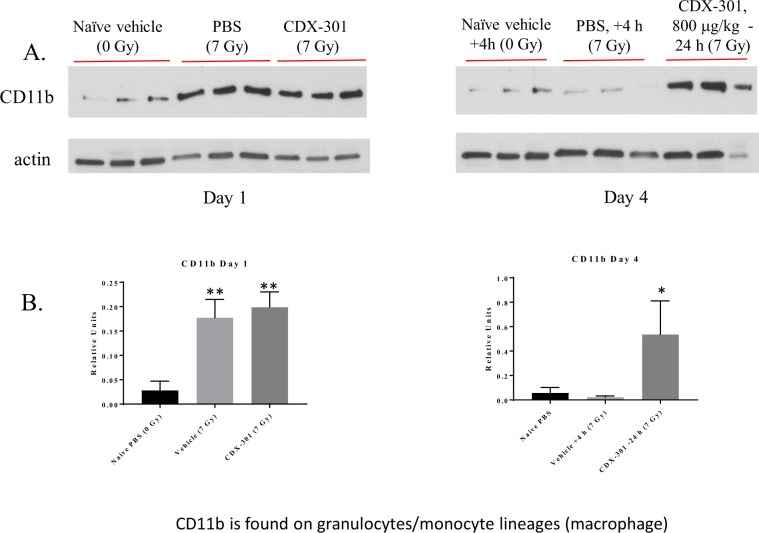
CDX-301 produces changes in splenic mononuclear cells. CDX-301 was administered 24 h prior to 7 Gy γ-radiation. The presence of CD11b+ macrophages (**A**) was determined by Western blot and quantified (**B**). *p ≤ 0.03; **p ≤ 0.001.

IL-2 and IFN-γ, in addition to T cell activation, activates macrophages. Based on this, we determined the percentage of total and activated macrophages under our experimental conditions (Table 1). We observed an IR mediated increase in the percentage of CD11b+ macrophages, both by flow cytometry and by Western blot (Fig. 11). The increase in CD11b was confirmed by Western blot (Fig. 11A,B). CDX-301 administered 4 h after IR prevented the IR-mediated increase in macrophage percentage and activation. The percentage of NK cells in the spleen increased in both IR and IR + CDX-301 (−24 h) groups, but mice treated with CDX-301 after radiation (T = +4 h) maintained a percentage of NK cells similar to spleens from non-irradiated mice (Table 1).

## Discussion

This work demonstrates the radioprotective and radiomitigative efficacy of CDX-301, the soluble extracellular domain of the native Flt3 ligand (Flt3L). All animals survived when the drug was given at a dose of 800 µg/kg 24 h before radiation. When the drug was given after radiation (4 h or 24 h), about 30% improved survival was accomplished compared to control group. Better results were achieved in terms of accelerated recovery from radiation-induced peripheral blood cytopenia when the drug was given prophylactically. Recovery from BM aplasia was also correlated to the blood cell recovery profile. Speed of restoration of sternal BM was higher in pre-TBI treated animals. Similarly recovery of BM stem and progenitor cells was accelerated when CDX-301 was given 24 h before radiation. The remarkable radioprotective efficacy of a single dose of CDX-301 was found to be superior compared to other sources of Flt-3L tested previously. Gratwohl *et al*. demonstrated the efficacy of recombinant human (rh) Flt-3L in white rabbits exposed to a lethal dose of TBI^32^. Other hematopoietic cytokines or growth factors were tested as potential radiation countermeasures administered either alone or in combination^2^. Although Neupogen, Neulasta, and Leukine are approved as a radiation countermeasures for ARS by FDA^5,6^, those are effective only when given after exposure. In addition, modest effects in animal survival from sublethal dose of TBI were observed with other cytokines, interleukin-3 (IL-3), interleukin-6 (IL-6), and granulocyte colony stimulating factors (GM-CSF)^33–35^. Orally administered IL-11 was found to be effective in mice exposed to a lethal dose of TBI. IL-11 was given at various daily doses starting at 24 h post-TBI, which continued up to 5 days. IL-11 treated animals had significant improvement in crypt survival as well as mucosal surface area and reduced bacterial translocation in liver^22,36–38^.

BM aplasia after TBI is the major consequence of radiation-induced apoptosis in hematopoietic stem and progenitor cells. An *in vitro* study indicated the anti-apoptotic effects of Flt-3L by promoting the maintenance of hematopoietic progenitor cells. In our study, we have shown there occurs a significant reduction in radiation-induced apoptosis in bone marrow sternum of CDX-301 treated animals compared to vehicle control.

We have shown that sublethal irradiation altered the splenic T cell population on day one, based on CD3 expression. Our data identified both high and low CD3 expression in splenocytes that are also CD4+. This population was also seen in splenocytes from mice treated with CDX-301 24 h prior to TBI. A transient increase in splenocytes, particularly T cells, was previously reported after low dose radiation exposure^39^. We hypothesize that the mixed populations of CD3^hi^ CD4^hi^ and CD3^lo^ CD4^lo^ cells may represent both unstimulated and stimulated Th cells, but not Tc cells. It was shown that stimulated T cells are more radioresistant than unstimulated cells, with a higher level of apoptosis seen in unstimulated T cells^40^. This CD3^lo^ CD4^lo^ population may also be regulatory T cells (Tregs), and the maintenance of Treg after radiation have been reported^41^. Although we did not fully examine both high and low CD3+ expressing cell populations, we showed that ionizing radiation (IR) caused increased expression of Th1 cytokines IL-2 and IFN-γ, and that treatment with CDX-301 prior to IR did not attenuate this response. In contrast, splenocytes from mice treated with CDX-301 4 h after IR displayed CD3 expression similar to that of non-irradiated, non-drug treated mice. IL-2 and IFN-γ levels remained at that of control splenocytes when CDX-301 was administered after IR exposure. In addition, Tc cells (CD3+ CD8+) were reduced in irradiated mice that were treated with CDX-301 prior to exposure, but not in mice treated with CDX-301 after exposure. Additional studies to characterize the CD3+ splenocyte population, including activation status and function, would provide insight into the status of the immune system after IR. It is important to note that the effectiveness of CDX-301 on T cell protection was transient. By day 7 similar decreases in the percentages of both Th and Tc cells were seen in all irradiated groups (data not shown). The radiation effects on both NK and macrophage percentages were lost by day 7, with the exception of the IR + CDX-301 (T = −24 h) treated mice. It is also interesting to note that although immune cell preservation was found in mice treated with CDX-301 after IR, improved survival was seen when animals were treated either before or after radiation exposure.

Macrophages are considered radioresistant, and may be a therapeutic target against radiation injury^42^. Our data reveals an increased percentage of total and activated (CD69+) macrophages in spleens from irradiated mice, as well as mice treated with CDX-301 24 h prior to radiation. CD69 is known as an early activation marker but is also involved in an inflammatory response. It was recently shown that CD11b CD69+ cells may interact with CD4+ T cells and influence proliferation, but not activation^43^. Following the pattern seen with T cells, mice treated with CDX-301 4 h after radiation maintain macrophage populations similar to that of non-irradiated mice.

Flt3L functions include the expansion of DC, but this was not clearly seen in our study. In the spleen, we detected limited protection of CD11c + DC in irradiated mice pre-treated with CDX-301 compared to vehicle-treated, irradiated mice. This was seen one and four days after radiation exposure, but the levels of protection were not significant. Anandasabapathy *et al*. reported increased DC after multiple injections of CDX-301 in the clinic^9^. The modest DC protection observed may be enhanced with multiple injections of CDX-301. In addition, DC were not analyzed in circulating blood cells in this study.

Taken together, we determined that CDX-301 administration induced altered radiation-induced immune responses, depending on the time of administration. We hypothesize that CDX-301 administration prior to radiation may engage immune activation and administration after radiation may engage immune protection. Further studies are needed to prove this hypothesis.
